# Feeding and Manual Brushing Influence the Release of Oxytocin, ACTH and Cortisol Differently During Milking in Dairy Cows

**DOI:** 10.3389/fnins.2022.671702

**Published:** 2022-03-14

**Authors:** Ewa Wredle, Kerstin Svennersten-Sjaunja, Lene Munksgaard, Mette S. Herskin, Rupert M. Bruckmaier, Kerstin Uvnäs-Moberg

**Affiliations:** ^1^Department of Animal Nutrition and Management, Swedish University of Agricultural Sciences, Uppsala, Sweden; ^2^Department of Animal Science, AU-Foulum, Aarhus University, Tjele, Denmark; ^3^Veterinary Physiology Vetsuisse Faculty, University of Bern, Bern, Switzerland; ^4^Department of Animal Environment and Health, Swedish University of Agricultural Sciences, Skara, Sweden

**Keywords:** cutaneous sensory nerves, afferent vagal nerves, feeding, brushing, oxytocin, dairy cows, milk yield, cortisol

## Abstract

**Aim:**

This study aimed to examine the effects of feeding or abdominal brushing on the release of the hormones oxytocin, ACTH and cortisol during milking in dairy cows.

**Methods:**

Twelve cows in early lactation were used (2 × 2 factorial experimental design), testing the effects of two types of sensory stimulation during milking over a 3 day period; feeding concentrate or manual abdominal brushing (1 stroke/s). Blood samples for hormone analyses were collected at time at −15, −1, 0 (onset of cluster), every min for 8 min, at 10, 12, 14, 16, 30, and 60 min. Hormone levels were assayed and AUC was calculated.

**Results:**

Milking was associated with an immediate and significant rise of oxytocin. When milking was combined with feeding, significantly higher levels of oxytocin were observed at 2 and 4 mins (*p* < 0.05). No effect of brushing on oxytocin levels was observed. Milking alone was associated with a significant rise of ACTH levels. Feeding in connection with milking reduced the immediate rise of ACTH levels (*p* < 0.05) and AUC (*p* < 0.02), whereas no effects of brushing were found. Milking caused a progressive rise of cortisol levels. Concomitant feeding did not influence cortisol levels, whereas brushing significantly decreased cortisol levels at 1, 5 and 14 mins after onset of milking (*p* < 0.05).

**Conclusion:**

Feeding increases oxytocin release in response to milking and decreases ACTH levels. Abdominal brushing did not influence these variables, but decreased cortisol levels. These data demonstrate that activation of afferent vagal nerve fibres and of cutaneous sensory nerves originating from the abdominal skin in front of the udder influence milking related hormone release differently.

## Introduction

The data from the present study represent a subset of results from a study in which the role of feeding and brushing of the abdominal area during milking on the release of hormones released during milking and on milk yield was studied. In this study, we focussed on the effect of feeding and brushing during milking on the milking induced release of oxytocin, ACTH and cortisol.

Oxytocin, which is produced in the supraoptic (SON) and paraventricular (PVN) nuclei of the hypothalamus, is released into the circulation from the posterior pituitary and also into the brain from oxytocinergic nerves originating in the PVN. Oxytocin released within the brain stimulates different types of social interactive behaviours and bonding, induces calmness, increases nociceptive thresholds and induces anti-stress like effects such as a decrease of the activity of the HPA axis and of the sympathetic nervous system, which is reflected by, e.g., reduced plasma cortisol concentrations, blood pressure and heart rate (as reviewed by Uvnäs-Moberg, 1998, 2014, 2015; Uvnäs-Moberg and Petersson, 2005). Specific subpopulations of oxytocin neurons are involved in different effects induced by oxytocin (Eliava et al., 2016; Grinevich and Stoop, 2018; Takahashi, 2021).

Oxytocin is released into the circulation in response to milking to induce milk ejection in dairy cows (for review see Bruckmaier and Blum, 1998). Both machine milking and calf’s suckling of the udder induces oxytocin release although studies have demonstrated a greater oxytocin release in cows when suckled compared with machine milking (e.g., Bar-Peled et al., 1995; Lupoli et al., 1999). In addition, social interactive behaviours and anti-stress effects are induced in connection with milking suggesting that oxytocin is also released from neurons originating within the brain during milking (Johansson et al., 1999a,b).

In rats, stroking of the abdomen increases the release of oxytocin into the brain and into the circulation and induces multiple oxytocin-linked effects such as increased nociceptive threshold and anti-stress effects *via* activation of sensory nerves originating in the skin of the abdomen (Uvnäs-Moberg and Petersson, 2011; Uvnäs-Moberg et al., 2015). Also in cattle, abdominal stroking induces anti stress affects, as heart rate is decreased (unpublished data). In addition, it increases the acceptance of and willingness to approach humans (Schmied et al., 2008) suggesting an oxytocin-mediated effect on social interactive behaviour.

We hypothesised that not only feeding but also abdominal brushing during milking could influence the release of oxytocin, ACTH and cortisol released during milking.

## Materials and Methods

### Animals and Management

The experiment was performed on 12 Swedish Red dairy cows at Kungsängen’s Research Centre, Uppsala, Sweden. The Uppsala Local Ethics Committee had approved the experiment Ref C271/3. The cows were in lactation number 1–4, in lactation week 9–24 (average 13 week), and had a daily milk yield of 26–43 kg (average 39 kg) at the beginning of the experiment. The cows were housed in individual tie-stalls and fed grass silage and concentrates according to their individual requirements based on the Swedish feeding recommendations (Spörndly, 2003).

The cows were machine milked twice a day; morning milking started at 06:15 h and evening milking at 17:00 h. The milking machine was an Alfa-Laval DuoVac (DeLaval, Tumba, Sweden) with the vacuum level at 45 kPa. Before the cluster was attached, the udder was cleaned with paper towel and control milk drawn from each udder quarter. The cluster was taken off, as soon as low-vacuum (33 kPa) appeared.

### Experimental Design

A 2 × 2 factorial design was used to test effects of feeding 2.1 kg concentrate and manual abdominal brushing one stroke per second during milking. All cows received all treatments in a balanced order. The experimental treatments were initiated from the start of pre-stimulation of the udder and brushing was terminated when the milking unit was detached. The treatments were performed for 3 days and included morning as well as evening milking. Data were collected during the morning milking on the third day of treatment.

### Blood Sampling and Assays

A semi-permanent catheter was inserted into a jugular vein of each cow 4 days before the first blood samples were collected. When not in use, the catheters were filled with Heparin (25 IU/ml, Lövens Läkemedel AB, Malmö, Sweden) dissolved in 0.9% NaCl. Blood was collected at 15 min and at 1 min before the onset of the cluster, i.e., start of pre-stimulation and treatments. In addition, samples were taken at time 0 (onset of cluster), and every minute during a period of 8 min as well as at 10, 12, 14, 16, 30, and 60 min after the onset of milking. At each sampling, a total of 20 ml of blood was drawn into two 10-ml EDTA tubes provided with Trasylol^®^ (400 IU/ml; Bayer Leverkusen, Germany) and immediately stored on ice. Within 1 h after blood collection, samples were centrifuged (10 min at 1,800 × g), plasma divided into four tubes and stored at –20°C. Plasma was analysed for oxytocin concentrations using radioimmunoassay, according to Schams (1983). The within-assay coefficient of variation (CV) varied from 5.9 to 7.8% and the between-assay CV from 11.2 to 16.9% in samples with high (17.2 ± 1.9 pmol/l) and low (1.6 ± 0.3 pmol/l) oxytocin concentration, respectively. Cortisol was analysed with an enzyme immunoassay as described by Sauerwein et al. (1991). The mean recovery after extraction was 77.5 ± 8.6%. Inter-assay coefficient of variation was 12.5. EDTA-stabilised plasma samples were assayed for ACTH using a modified version of the time resolved fluoro-immunometric assay (TRIFMA) initially developed for human blood samples by Dobson et al. (1987). Small modifications allowed for automated running of the assay on an automated analyser (AutoDELFIA 1235, Wallac Oy, Turku, Finland), thus the incubation temperature was 25°C instead of 4°C as used by Dobson et al. (1987). Calibration standard curves were prepared in serum from a cow pre-treated with dexamethasone. Otherwise, performance characteristics of the modified assay were similar to the original assay.

### Statistical Analysis

**FIGURE 1:**
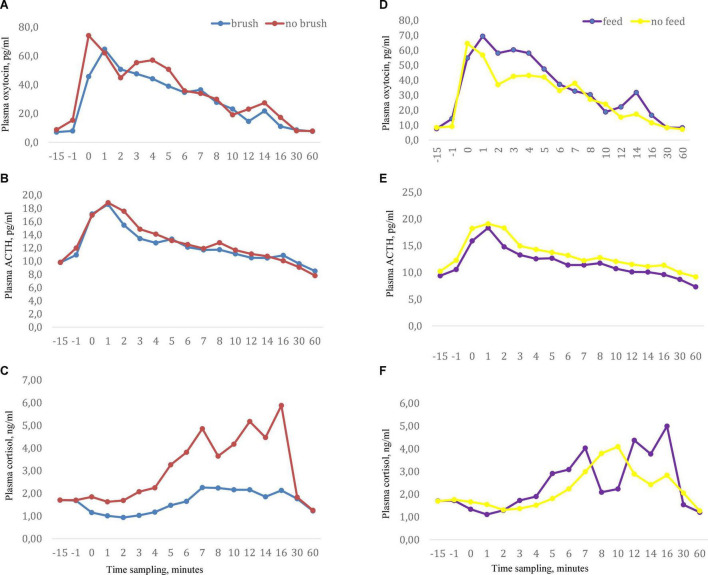
Milking related plasma concentrations of **(A)** oxytocin, **(B)** ACTH, and **(C)** cortisol when cows were exposed to the sensory treatments abdominal brushing or no brushing and **(D–F)** with stimuli feeding of concentrates or no feed during milking. Data showed as arithmetic mean values. Manual pre-stimulation started at time -1 and onset of milking cluster occurred at time 0, *n* = 11.

The Mixed procedure of SAS 9.1 (SAS, 2002) was used to analyse the data. To evaluate the effect of treatment on the hormone concentration, three response areas from area under curve (AUC) were calculated and AUC_1_ covered 0–10 mins, AUC_2_ covered 0–30 mins, and AUC_3_ covered 0–60 mins. The statistical model included the fixed effects of feeding and abdominal brushing, sampling time, experimental period, the interactions between sampling time × abdominal brushing and feeding, as well as the random effect of cow. Data was transformed to a logarithmic scale to obtain normal distribution before the statistical analyses. Results are presented as least square means with SE. To show the hormone patterns of the different stimuli we display arithmetic mean values in Figure 1. Mean differences between treatment effects were declared significant at *P* < 0.05.

## Results

### Plasma Concentration of Oxytocin

Basal (pre-milking) oxytocin concentrations did not differ across the treatments and ranged from 7.1 to 8.8 pg/ml. Milking gave rise to an increase in plasma oxytocin above basal levels immediately after attachment of the cluster and peak oxytocin levels were reached at 2–4 min after initiation of milking (Figure 1). When milking was combined with feeding, a significantly higher oxytocin concentration was obtained at 3 min (*P* < 0.05) and a tendency to higher levels was obtained at 4 min after start of milking compared to milking alone). No effects of abdominal brushing or feeding on AUC oxytocin levels were found (Table 1).

**TABLE 1:** Milking related plasma concentrations of oxytocin, ACTH and cortisol in dairy cows exposed to feeding or abdominal brushing during milking.

	Brush	No brush	*P*-value	Feed	No feed	*P*-Value
***AUC_1_ (0–10 min)***						
Oxytocin	382.6 ± 62.9	406.7 ± 64.0	0.73	430.6 ± 62.9	358.7 ± 64.0	0.31
ACTH	127.7 ± 15.1	137.9 ± 15.0	0.30	124.1 ± 15.1	141.5 ± 15.0	0.08
Cortisol	14.9 ± 7.9	28.2 ± 8.0	0.07	21.2 ± 7.9	22.0 ± 8.0	0.91
***AUC_2_ (0–30 min)***						
Oxytocin	624.1 ± 103.6	696.3 ± 105.2	0.51	731.7 ± 103.6	588.6 ± 105.2	0.20
ACTH	330.5 ± 33.0	328.5 ± 33.2	0.93	301.7 ± 33.0	357.3 ± 33.2	0.02
Cortisol	54.3 ± 40.2	106.7 ± 40.8	0.22	87.5 ± 40.2	73.5 ± 40.8	0.71
***AUC_3_ (0–60 min)***						
Oxytocin	867.5 ± 111.1	923.1 ± 112.9	0.64	971.4 ± 111.1	819.2 ± 112.9	0.20
ACTH	590.4 ± 56.6	543.0 ± 57.4	0.40	530.0 ± 56.6	603.4 ± 57.4	0.19
Cortisol	98.3 ± 49.9	148.6 ± 50.6	0.28	125.7 ± 49.9	121.5 ± 50.6	0.92

*Values represent area under the curve (AUC) during different time periods and least square means ± SEM, n = 11.*

### Plasma Concentration of ACTH

Basal (pre-milking) levels of plasma ACTH were similar across the treatments and ranged from 9.3 to 10.3 pg/ml. Milking increased the levels of ACTH and peak values were reached at approximately 2 min after initiation of milking (Figure 1). Feeding during milking lowered the plasma concentration of ACTH in comparison to milking without feeding. ACTH levels were significantly lower at the onset of milking and at 2, 3, 4, and 5 min after onset of milking (*P* < 0.05) in the feeding group (Table 2). In addition, the values of the response areas were lower when milking was combined with feeding for AUC_1_ (*P* = 0.08) and AUC_2_ (*P* = 0.02) while no difference between treatments was observed on AUC_3_ (Table 1). No effects of abdominal brushing on the levels of ACTH were found.

**TABLE 2:** Least squares means and SE in logarithmic transformed values of the hormone oxytocin, ACTH and cortisol with treatments feeding no feeding during milking and abdominal brushing or no brushing during milking.

Time, min	−15	−1	0	1	2	3	4	5	6	7	8	10	12	14	16	30	60	SE
Oxytocin, feed	1.85	2.23	3.65	3.90	3.85	3.95^a^	3.86	3.71	3.53	3.41	3.31	2.92	2.86	2.88	2.52	2.06	2.03	0.08
Oxytocin, no feed	1.93	1.97	3.26	3.64	3.40	3.48^b^	3.50	3.46	3.34	3.33	3.18	3.05	2.63	2.66	2.40	2.02	1.89	0.09
Oxytocin, brush	1.89	2.02	3.15	3.71	3.70	3.66	3.63	3.49	3.47	3.39	3.27	3.03	2.61	2.73	2.38	2.10	1.97	0.08
Oxytocin, no brush	1.88	2.17	3.77	3.83	3.55	3.77	3.75	3.67	3.39	3.35	3.23	2.94	2.88	2.82	2.54	1.98	1.94	0.08
ACTH feed	0.93	0.96	1.14^a^	1.18	1.12^a^	1.07^a^	1.05^a^	1.04^a^	1.02	1.02	1.03	0.98	0.96	0.97	0.95	0.91	0.82	0.05
ACTH no feed	0.98	1.05	1.21^b^	1.24	1.22^b^	1.15^b^	1.13^b^	1.11^b^	1.10	1.04	1.09	1.06	1.04	1.02	1.03	0.98	0.92	0.05
ACTH brush	0.95	1.00	1.19	1.22	1.15	1.09	1.07	1.08	1.05	1.04	1.04	1.00	0.99	0.98	0.99	0.95	0.88	0.05
ACTH no brush	0.96	1.00	1.15	1.19	1.19	1.13	1.10	1.07	1.07	1.02	1.07	1.03	1.00	1.01	0.98	0.93	0.85	0.05
Cortisol feed	0.00	0.00	−0.11	−0.47	−0.35	−0.19	−0.07	0.26	0.25	0.51	0,38	0.48	0.52	0.21	0.38	−0.12	−0.31	0.22
Cortisol no feed	0.22	0.12	−0.09	0.03	−0.08	0.00	0.05	0.12	0.35	0.45	0.68	0.71	0.64	0.47	0.50	0.23	−0.23	0.23
Cortisol brush	0.07	0.03	−0.33	−0.55^a^	−0.47	−0.34	−0.23	−0.08^a^	0.07	0.36	0.44	0.27^a^	0.38	0.06^a^	0.33	−0.07	−0.47	0.22
Cortisol no brush	0.16	0.09	0.14	0.09^b^	0.04	0.15	0.22	0.47^b^	0.54	0.60	0.93	0.79^b^	0.64	0.53^b^	0.53	0.17	−0.08	0.23

*Statistical analyses were performed within hormones and between the treatments feed no feed; brush no brush. Means within respective hormones and between feeding no feeding or brushing no brushing with different letters are significantly different (P < 0.05).*

### Plasma Concentration of Cortisol

In general the plasma levels of cortisol were low. Basal cortisol levels were similar across the treatments and ranged from 1.7 to 1.8 ng/ml. Milking by itself gave rise to an increase in plasma cortisol and peak levels were reached at the end of milking or just after the milking were finished (Figure 1). Feeding did not influence milking induced cortisol levels. However, when milking was combined with brushing the plasma cortisol levels were significantly lower (*P* < 0.05) at 1, 5, and 12 min after the onset of milking compared to milking with no brushing (Table 2). In addition, when milking was combined with brushing the cortisol AUC_1_ tended to be lower (*P* = 0.07) when compared to values obtained for milking with no brushing (Table 1).

## Discussion

Milking was accompanied by a release of the hormones oxytocin, ACTH and cortisol. The milking induced hormonal pattern was modified, when milking was combined with feeding or brushing. Feeding tended to increase oxytocin levels whereas ACTH levels were significantly decreased. Cortisol levels alone were decreased by brushing.

Interestingly, feeding in combination with milking induced small but significantly higher levels of oxytocin at 3 min and a tendency to increased levels 4 min after the onset of milking as well as numerically increased response areas, when compared to milking without feeding. These data are in line with previous studies (Svennersten et al., 1995; Johansson et al., 1999a,b). The reinforced increase in oxytocin levels caused by feeding is mediated by an enhanced afferent vagal nerve activity. When food is present in the gastrointestinal tract, gastrointestinal hormones, such as cholecystokinin (CCK) are released, which promote afferent vagal nerve activity (Uvnäs-Moberg, 1994). The importance of the link between the gastrointestinal tract and milk production is demonstrated in studies by Eriksson et al. (1994) performed on lactating rats. The data show that the suckling-related release of oxytocin and prolactin is strongly dependent on intact vagal nerves, since suckling-related oxytocin and prolactin responses were almost abolished in rats subjected to vagotomy and the weight gain of the pups was severely impaired, when no milk was produced and ejected. These data are clearly in line with the findings of a feeding induced reinforcement of the milking-related oxytocin release and thereby milk production caused by a feeding-induced increase of afferent vagal nerve activity.

The role of ACTH in the control of milking-induced cortisol secretion has been questioned, since no release of ACTH was recorded in response to milking in a previous study (Tancîn et al., 2000). The present data, however, showing a clear rise of ACTH in response to milking suggest that ACTH does play an important role for the release of cortisol during milking, since the rise of ACTH levels precedes the increase of cortisol levels.

The feeding treatment was associated by significantly lower levels of plasma ACTH during milking. The observed increase in oxytocin levels and decrease in ACTH levels in the fed cows may be linked to each other. In rats oxytocin released within the PVN exerts a direct inhibitory effect on the release of corticotropin-releasing factor (CRF) from the PVN, which controls the secretion of ACTH from the anterior pituitary (Neumann et al., 2000; Takahashi, 2021). In addition, oxytocin decreases ACTH secretion by an effect in the anterior pituitary (Pettersson et al., 1999; Uvnäs-Moberg, 2015). Given that the secretion of ACTH is controlled in a similar way in cows the decreased ACTH levels may be a consequence of effects of oxytocin in both the hypothalamus and anterior pituitary. We did not observe a decrease of cortisol levels, which would have been expected, given the decrease of ACTH levels, suggesting a complex action of the feeding stimulus on cortisol secretion.

Manual brushing of the abdomen did not influence milking-related oxytocin release. Brushing, of the abdomen, however, reduced cortisol levels compared to control, but did not affect ACTH levels. These findings were somewhat unexpected, since oxytocin is released both into the circulation and into the CSF in response to massage-like stroking of the abdomen in rats (Ågren et al., 1995).

The lack of an increase of oxytocin levels and decrease of ACTH levels in response to manual abdominal brushing during milking suggests that different effect patterns are induced following by suckling/afferent stimulation of vagal nerves in connection with feeding and following stimulation of sensory nerves in connection with brushing of the abdomen in dairy cows.

Similar observations regarding differences in the control of cortisol release have been made in humans. Oxytocin is released in response to suckling in breastfeeding women in order to promote milk ejection. At the same time the activity of the HPA axis is decreased as levels of ACTH decreases (Handlin et al., 2009). This decrease of ACTH levels seems to be mediated by an oxytocin mediated decrease of CRF secretion in the PVN (Uvnäs-Moberg et al., 2021; Takahashi, 2021). In addition ACTH levels may be decreased *via* oxytocin secreted into the anterior pituitary. Cortisol levels are decreased in response to breastfeeding (Handlin et al., 2009), which is also associated with a substantial decrease in blood pressure and an increased function of the endocrine system of the gastrointestinal tract, indicating that sympathetic nervous tone is decreased and parasympathetic nervous tone is increased in response to suckling. Social interactive behaviour is also increased during breastfeeding. These effects should be mediated by oxytocinergic fibres emanating in the PVN (Uvnäs-Moberg et al., 2020).

Oxytocin is, however, not released into the circulation in response to skin-to-skin contact between mother and newborns *per se* after birth (as measured with RIA) and there is no change in the levels of ACTH in humans. Yet the sensory stimulation of the skin of the front side in mothers in connection with skin-to-skin contact between mothers and babies is associated with decreased cortisol levels (Handlin et al., 2009). In addition, other stress relieving effects are induced and social interaction is stimulated (for a review see of these effects see Uvnäs-Moberg et al., 2021).

As mentioned above the SON and PVN contain magnocellular neurons that transfer oxytocin to the posterior pituitary and the circulation. In addition the PVN contains several types of parvocellular neurons, which influence basic physiological and behavioural functions such as the function of the autonomic nervous system (Sofroniew, 1980; Buijs, 1983). Oxytocin release into the circulation does not always parallel oxytocin release into the brain, suggesting that different aspects of the oxytocin system can be activated separately (Takahashi, 2021). It is therefore possible, but remains to be proven, that stroking of the skin in front of the udder in the cow, just like skin-to-skin contact between mother and infant and ventral stimulation of the skin in rats only activate a subgroup of the oxytocin neurons, e.g., these neurons involved in the stimulation of social interaction/maternal behaviour and also in reducing stress levels by decreasing the activity of the sympathetic nervous system and the sympathoadrenal system.

Different effects obtained by suckling, afferent vagal stimulation and by sensory stimulation of skin on the ventral side of the abdomen suggests that the function of the adrenal cortex can be regulated at two levels; by ACTH released from the pituitary and also by sympathetic nervous tone. Both these processes should be influenced by oxytocin, but *via* separate mechanisms. The former one could involve an effect of magnocellular oxytocin neurons that inhibit CRF secretion from the PVN and to some extent directly decrease the release of ACTH from the anterior pituitary and one that does not involve the classical HPA axis, but oxytocinergic nerves emanating from the PVN that control autonomic nervous function (Uvnäs-Moberg et al., 2021).

As mentioned above there is a dissociation between ACTH and cortisol secretion during skin-to-skin contact between mother and infant. Those data suggests that stimulation of cutaneous afferents from the front side may modify cortisol without influencing ACTH secretion in humans. The mechanism behind this effect can at present only be speculated upon. It is, however, possible that the inhibition of cortisol release from the adrenal gland induced by stimulation of the skin on the front side occurs *via* a decreased activity of sympathetic nervous tone. The function/binding of the ACTH receptors in the adrenal gland is enhanced by the activity in the sympathetic nervous system. Brushing of the abdomen in humans and rats may decrease the activity in the branches of the sympathetic nerves, innervating the adrenal cortex, which may lead to a decreased function of the ACTH receptors and a subsequent decrease of cortisol secretion, in spite of unchanged ACTH levels (Stachowiak et al., 1995; Sato et al., 1997; Pettersson et al., 1999). Oxytocin released from oxytocinergic nerves projecting from the PVN to the brainstem may participate in this effect. Although it remains a speculation a similar mechanism may be activated in response to brushing of the skin in front of the udder of the cow.

Perhaps the stress reducing effects induced by stroking or touching of the skin represents a more original and primitive stress regulating mechanism than does the one involving the HPA axis.

In conclusion, feeding increases oxytocin release in response to milking and decreases ACTH levels. Abdominal brushing did not influence these variables, but decreased cortisol levels. These data demonstrate that activation of afferent vagal nerve fibres and of cutaneous sensory nerves, originating from the abdominal skin in front of the udder, influence milking related hormone release differently, perhaps by activating different populations of oxytocin neurons in the SON and PVN.

## Data Availability Statement

The original contributions presented in the study are included in the article/supplementary material, further inquiries can be directed to the corresponding author.

## Ethics Statement

The animal study was reviewed and approved by Uppsala Local Ethics Committee.

## Author Contributions

EW organised the database and wrote the first draft of the manuscript. EW and LM performed the statistical analysis. KU-M, LM, and MH wrote sections of the manuscript. All authors contributed to the conception, design of the study, read, and approved the submitted version.

## Conflict of Interest

The authors declare that the research was conducted in the absence of any commercial or financial relationships that could be construed as a potential conflict of interest. The reviewer HJ is currently organising a Research Topic with the author KU-M.

## Publisher’s Note

All claims expressed in this article are solely those of the authors and do not necessarily represent those of their affiliated organizations, or those of the publisher, the editors and the reviewers. Any product that may be evaluated in this article, or claim that may be made by its manufacturer, is not guaranteed or endorsed by the publisher.
